# Clinical Effects Observation of ICL Implantation With Personalized Surgically Induced Astigmatism for Correcting Low to Moderate Astigmatism With Myopia in Patients

**DOI:** 10.1155/joph/6649909

**Published:** 2025-09-30

**Authors:** Ting Huang, Siyi Bao, Ke Li

**Affiliations:** Department of Ophthalmology, Army Medical University Daping Hospital, Chongqing, China

**Keywords:** astigmatism, implantable Collamer lens, surgically induced astigmatism, target-induced astigmatism

## Abstract

**Objective:**

To evaluate the clinical effectiveness of personalized surgically induced astigmatism (SIA) combined with ICL implantation for correcting low to moderate astigmatism with myopia in patients.

**Methods:**

A retrospective, noncomparative, noninterventional case series. The study included 55 myopic patients (87 eyes) with low to moderate astigmatism who underwent ICL implantation. All patients received a transparent corneal incision at the corneal steep axis, introducing personalized SIA. Preoperative and postoperative visual acuity, intraocular pressure (IOP), refractive diopter, corneal curvature, corneal astigmatism (CA), astigmatism axis, and aberrations were evaluated. Preoperative and postoperative data changes were analyzed, and CA vector analysis was performed.

**Results:**

The uncorrected distance visual acuity (UDVA) at 1 week and 6 months postoperatively were similar to the preoperative corrected distance visual acuity (CDVA) (*p*1 = 0.870, *p*2 = 0.043), and better than the estimated corrected distance visual acuity (EsCDVA) (*p*1 < 0.001, *p*2 < 0.001). The postoperative UDVA remained stable over time (*p*=0.054). The ocular refractive astigmatism (RA) decreased by −0.43 D and approximately 51.81% (*p* < 0.001) at 1 w postoperatively, and by −0.32 D and approximately 38.55% (*p* < 0.001) at 6 m postoperatively. CA decreased by −0.38 D and approximately 30.65% (*p* < 0.001) at 1 w postoperatively, and by −0.27 D and approximately 21.77% (*p* < 0.001) at 6 m postoperatively. The postoperative regression in RA was approximately −0.11 D (*p*=0.011), and in CA, it was approximately −0.11 D (*p*=0.094). The postoperative total corneal aberrations and low-order aberration (LOA) were decreased (*p* < 0.05, *p* < 0.05), and high-order aberration (HOA) was increased (*p* < 0.05). As time progressed postoperatively, the corrective results tended to regress. The postoperative vertical coma was decreased (*p* > 0.05), and the horizontal coma and the spherical aberration were increased (*p* > 0.05, *p* > 0.05). As time progressed postoperatively, the postoperative variation tended to be obvious. The SIA values at 1 week and 6 months postoperatively were −0.41 D ∗ 89 (mean −0.52 D) and −0.28 D ∗ 88 (mean −0.42 D), the target induced astigmatism (TIA) values were −0.25 D ∗ 87 (mean −0.43 D) and −0.23 D ∗ 87 (mean −0.32 D), and the correlations between TIA and SIA were *y* = 0.44*x* + 0.33, *R*^2^ = 0.24 and *y* = 0.32*x* + 0.31, *R*^2^ = 0.19. The difference vector (DV) values were −0.16 D ∗ 2 (mean −0.50 D) and −0.05 D ∗ 4 (mean −0.41 D). The postoperative correction index (CI) values were all greater than 1, indicating mild overcorrection. Notably, the effect of overcorrection was more pronounced at 1 w postoperatively than 6 m. The index of success (IOS) values were 1.56 and 4.6.

**Conclusion:**

ICL implantation with personalized SIA can achieve effective surgical outcomes for myopic patients with moderate to low astigmatism. However, as time progressed postoperatively, some corrective results tended to regress.

## 1. Introduction

Astigmatism is a common refractive error that often occurs in conjunction with myopia. It generally refers to ocular refractive astigmatism (RA), which is the vector sum of corneal astigmatism (CA) and ocular residual astigmatism (ORA) [1]. Astigmatism is a low-order aberration (LOA) that affects overall visual acuity, increases fatigue, and decreases visual quality. To achieve good surgical results, astigmatism correction should be considered during myopic surgery.

Myopic surgery comprises corneal refractive surgery and intraocular refractive surgery. The common intraocular refractive surgery is posterior chamber intraocular lens implantation. Implantable Collamer lens (ICL) is the most commonly used posterior chamber intraocular lens in clinical practice, including ICL and Toric ICL (TICL), which can correct myopia up to −18.0 D and astigmatism up to 6.0 D. It offers good surgical safety, a stable postoperative effect, superior visual quality, and night vision. Additionally, it is the reversible surgery for myopia correction [2]. TICL can correct myopia and astigmatism simultaneously. However, the significant effects of surgically induced astigmatism (SIA) are often overlooked in preoperative calculations, and the possibility of TICL astigmatism axis rotation (astigmatism increasing) persists postoperatively. The stability of TICL and residual astigmatism all affect postoperative outcomes, with the possibility of secondary surgical realignment. Therefore, there are certain limitations to TICL for myopia and astigmatism correction.

Clinical studies indicate that myopic patients with moderate to low astigmatism, especially for patients with astigmatism < 0.5 D [3], can achieve good vision without the need to correct astigmatism. By using SIA generated by surgical incisions, astigmatism may be corrected. Its clinical effectiveness has been fully demonstrated in cataract surgery [4–7], providing a theoretical basis for ICL implantation combined with personalized SIA correction of astigmatism. However, the current analysis of the astigmatism correction effectiveness of ICL implantation with personalized SIA is not comprehensive enough [8, 9]. By analyzing the postoperative effects and the changes in residual refractive error of ICL with personalized SIA correction, clinical guidance can be provided for selecting surgical options for patients with low to moderate astigmatism and myopia.

## 2. Objects and Methods

### 2.1. Objects

#### 2.1.1. Retrospective Analysis

Patients who underwent ICL implantation at Daping Hospital from June 2021 to May 2022 were selected, including 55 people (9 males, 46 females) and 87 eyes in total. All patients were informed preoperatively of the risks of ICL implantation and of personalized SIA correction for low to moderate astigmatism, and the impact of personalized SIA correction on postoperative vision. All of them expressed their understanding and agreed to the surgery by signing the surgery informed consent form. All patients were informed about the use of their preoperative and postoperative data for the study, and they expressed their agreement and signed the informed consent form. The study was approved by the hospital ethics committee [Medical Research Involving People Ethical Ratification No: 2023(54)]. All methods followed relevant guidelines and standard operating procedures.

##### 2.1.1.1. Inclusion Criteria

(1) Willingness to undergo myopic surgery; (2) 18–45 years old; (3) diopter remains stable for ≥ 2 years, with an increase of < 0.5 D within 2 years; (4) indications for ICL implantation; (5) ACD ≥ 2.80 mm; (6) ECD ≥ 2000 cells/mm^2^; (7) scotopic pupil diameter ≤ 7 mm; (8) Sph ≥ −3.0 D and −0.25 D ≤ Cyl ≤ −2.25 D; (9) Axial variance between CA and RA < 15°.

##### 2.1.1.2. Exclusion Criteria

(1) History of trauma or eye surgery; (2) obvious irregular astigmatism, pterygium, corneal scars, and other corneal lesions; (3) ocular inflammation such as uveitis; (4) anterior chamber angle stenosis; (5) eye diseases such as glaucoma, complicated cataracts, retinal disorders, and optic neuropathy; and (6) incomplete medical records.

### 2.2. Methods

#### 2.2.1. Preoperative Examination

All patients were examined by slit lamp for anterior segment, slit lamp combined with indirect funduscopy for fundus examination, standard logarithmic visual acuity chart for visual acuity examination, noncontact tonometer for intraocular pressure (IOP) examination, auto kerato-refractometer, and phoropter to record optometry results. Visante's anterior segment OCT (Carl Zeiss, Germany) was used for anterior segment condition and pupil diameter measurements, Pentacam anterior segment analysis system (Oculus, Germany) was used for corneal condition measurements, corneal endothelial counting was used for ECD measurements, IOLmaster was used for eye axis measurements, and UBM was used for ciliary sulcus morphology measurements. Visual acuity, IOP, computerized and comprehensive optometry, ACD, WTW, scotopic pupil diameter, corneal curvature(K1, K2), astigmatism axis, corneal aberration (total corneal aberration, LOA, high-order aberration (HOA), vertical coma, horizontal coma, and spherical aberration), and ECD were recorded.

#### 2.2.2. ICL Lens Selection

ICL sizes were available in four varieties: 12.1, 12.6, 13.2, and 13.7 mm, with optical diameters ranging from 4.9 to 5.8 mm. By using an online calculation formula provided by the STAAR company, the ICL lens size was determined by the refractive error, corneal curvature, ACD, WTW, STS, and the shape of the ciliary sulcus, which were referred to corneal topography, anterior segment OCT, and UBM [2].

#### 2.2.3. Surgical Methods

All patients were given antibiotic eye drops for 3 days to prevent infection preoperatively. They received corneal marking and adequate pupil dilation 1 h preoperatively. A 3-mm-wide steep-axis transparent corneal incision, matching the Pentacam, was made under surface anesthesia, 1-2 mm away from the corneal limbus. The ICL implantation was performed by the same physician who is qualified to perform this procedure, implanting EVO (V4c) Lens. Patients were given routine care including the use of antibiotics, corticosteroids, nonsteroidal anti-inflammatory drugs, and artificial tear eye drops postoperatively.

#### 2.2.4. Follow-Up Observation

All patients were followed up for at least half a year, and postoperative data at 1 week and 6 months were included in the study. For each review, visual acuity, IOP, optometry, corneal curvature (K1, K2), astigmatism axis, total corneal aberration, LOA, HOA, vertical coma, horizontal coma, and spherical aberration were included, and the examination methods were the same as those used preoperatively.

All the data were used for retrospective analysis by SPSS19.0 and statistical descriptions by mean ± SD. As the data satisfied normal distribution, paired *t*-test was used to test the differences between the preoperative and postoperative data, independent sample *t*-test was used to test the difference between the postoperative 1 w and the postoperative 6 m data. Otherwise, Wilcoxon signed-rank test would be used. For controlling inter-ocular symmetry, generalized estimating equations (GEE) analysis was used [10]. The statistical results were considered significant if *p* < 0.05.

Alpin's method was utilized to analyze the astigmatism vectors for the differences between preoperative and postoperative 1-week and 6-month periods. This analysis included the target-induced astigmatism (TIA) vector, SIA vector, difference vector (DV), correction index (CI), and index of success (IOS). CI = SIA/TIA. If CI > 1, it indicates overcorrection. If CI < 1, it indicates undercorrection. If CI = 1, it is neither undercorrected nor overcorrected. IOS = TIA/DV. The larger the IOS, the smaller the difference in astigmatism from the target.

## 3. Results

### 3.1. Preoperative Characteristics of Eyes With Moderate to Low Astigmatism and Myopia Patients (Table 1, Figure 1)

.

### 3.2. Transformation in Visual Acuity and Diopter at the Preoperative Period Versus Postoperative 1-w and 6-m Periods

**Figure 2:**
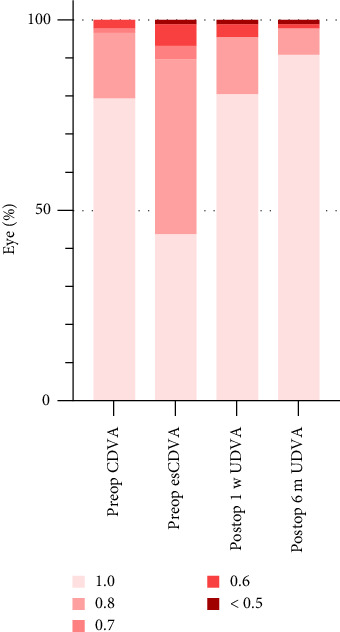
Visual acuity of preoperative versus postoperative 1 w and 6 m.

The uncorrected distance visual acuity (UDVA) at 1 week and 6 months postoperatively were similar to the preoperative CDVA (*p*1=0.870, *p*2=0.043), and it were better than the EsCDVA (*p*1 < 0.001, *p*2 < 0.001) (see Table 2, Figure 2). The postoperative UDVA remained stable over time (*p*=0.054), suggesting that ICL implantation with personalized SIA can achieve better postoperative visual acuity.

**Figure 3:**
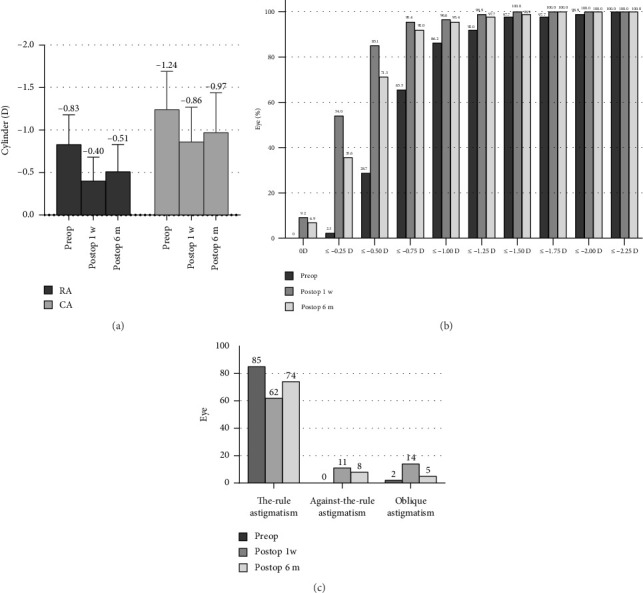
(a) Refractive cylinder of preoperative versus postoperative 1 w and 6 m. (b) Refractive cylinder of preoperative versus postoperative 1 w and 6 m. (c) Astigmatic axis of preoperative versus postoperative 1 w and 6 m.

Compared with the preoperative levels (see Table 2, Figure 3(a)), the RA decreased by −0.43 D and approximately 51.81% (*p* < 0.001) at postoperative 1 w, and by −0.32 D and approximately 38.55% (*p* < 0.001) at postoperative 6 m. The CA decreased by −0.38 D and approximately 30.65% (*p* < 0.001) at postoperative 1 w and by −0.27 D and approximately 21.77% (*p* < 0.001) at postoperative 6 m. The postoperative regression in RA was approximately −0.11 D (*p*=0.011), and in CA, it was approximately −0.11 D (*p*=0.094). At postoperative 1 w and 6 m, none of the eyes showed astigmatism of ≥ −2.5 D, 96.55% and 95.40% had astigmatism of ≤ −1.0 D, 85.06% and 71.26% had astigmatism of ≤ −0.5 D, and 9.20% and 6.90% had astigmatism of ≤ 0 D (see Figure 3(b)). These findings demonstrated that ICL implantation with personalized SIA can effectively correct astigmatism in the entire eye. However, there existed a regression in astigmatism correction over time postsurgery.

The-rule astigmatism accounted for approximately 97.70% at preoperative time, decreased to 71.26% at postoperative 1 w, and recovered to 85.06% at postoperative 6 m. There was no against-the-rule astigmatism at preoperative time, but it rose to 12.65% at postoperative 1 w and decreased to 9.20% at postoperative 6 m. Oblique astigmatism accounted for approximately 2.30% at preoperative time, rose to 16.09% at postoperative 1 w, and decreased to 5.74% at postoperative 6 m (see Figure 3(c)). These findings demonstrated that ICL implantation with personalized SIA can reduce astigmatism and change the astigmatism axis. However, as the operation time goes by, there exists a regression in astigmatism axial change.

### 3.3. Corneal Aberrations Tendency of Preoperative Versus Postoperative 1 w and 6 m

**Figure 4:**
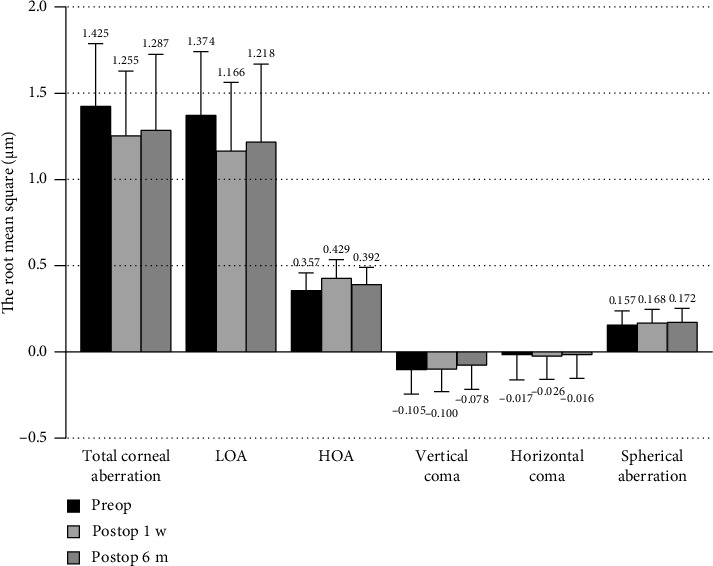
Corneal aberrations of preoperative versus postoperative 1 w and 6 m.

Compared with preoperative levels (see Table 3, Figure 4), the total corneal aberrations decreased by 0.17 (11.93%, *p* < 0.001) and 0.138 (9.68%, *p* < 0.001) at postoperative 1 w and 6 m, respectively, and the postoperative regression was 0.032 (*p*=1.000). The LOA decreased by 0.208 (15.14%, *p* < 0.001) and 0.162 (11.79%, *p* < 0.001) at postoperative 1 w and 6 m, respectively, the postoperative regression was 0.046 (*p*=0.524). The HOA increased by 0.072 (20.17%, *p* < 0.001) and 0.035 (9.80%, *p*=0.019) at postoperative 1 w and 6 m, respectively, and the postoperative change was 0.037 (*p*=0.003). The vertical coma decreased by −0.005 (4.76%, *p*=0.605) and −0.027 (25.71%, *p*=0.016) at postoperative 1 w and 6 m, respectively, and the postoperative regression was −0.022 (*p*=1.000). The horizontal coma increased by −0.009 (52.94%, *p*=0.623) and decreased by −0.001 (5.88%, *p*=1.000) at postoperative 1 w and 6 m, respectively, and the postoperative variation was −0.010 (*p*=0.446). The spherical aberration increased by 0.011 (7.01%, *p*=0.262) and 0.015 (9.55%, *p*=0.163) at postoperative 1 w and 6 m, respectively, and the postoperative variation was 0.004 (*p*=1.000). These findings indicated that ICL implantation with personalized SIA can effectively correct total corneal aberration and LOA, while also introducing HOA. Furthermore, it reduces vertical aberration and increases both horizontal aberration and spherical aberration. However, the changes observed in horizontal aberration and spherical aberration were not statistically significant. Additionally, as time progressed postoperatively, some corrective results tended to regress.

### 3.4. Vector Analysis of CA of Preoperative Versus Postoperative 1 w and 6 m (Figures 5, 6, 7)

**Figure 5:**
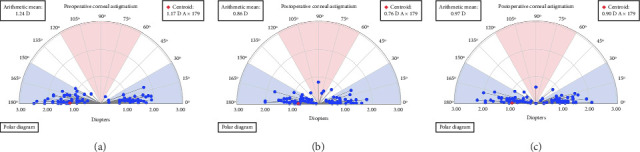
Corneal astigmatism of preoperative versus postoperative 1 w and 6 m. (a) Preop. (b) Postop 1 w. (c) Postop 6 m.

**Figure 6:**
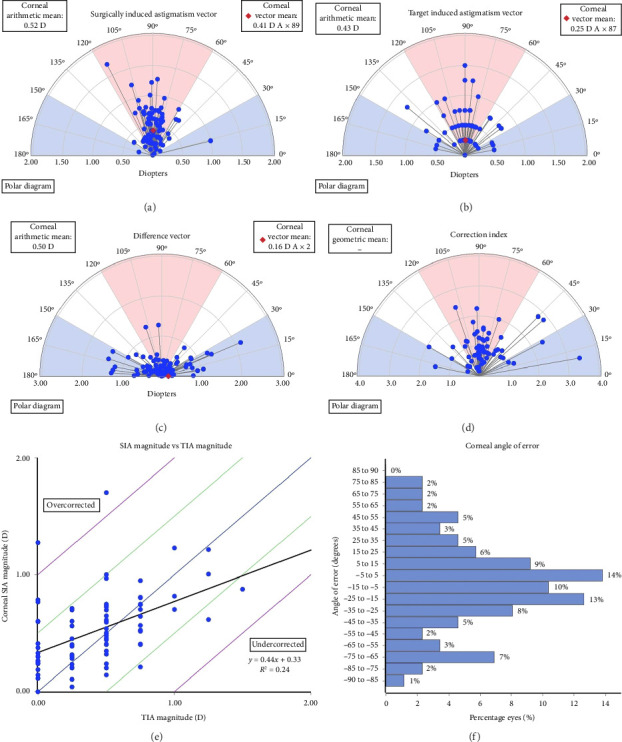
Single-angle polar plots at 1 week postoperative (a) SIA; (b) TIA; (c) DV; (d) CI; (e) SIA vs TIA; (f) Corneal angle of error.

**Figure 7:**
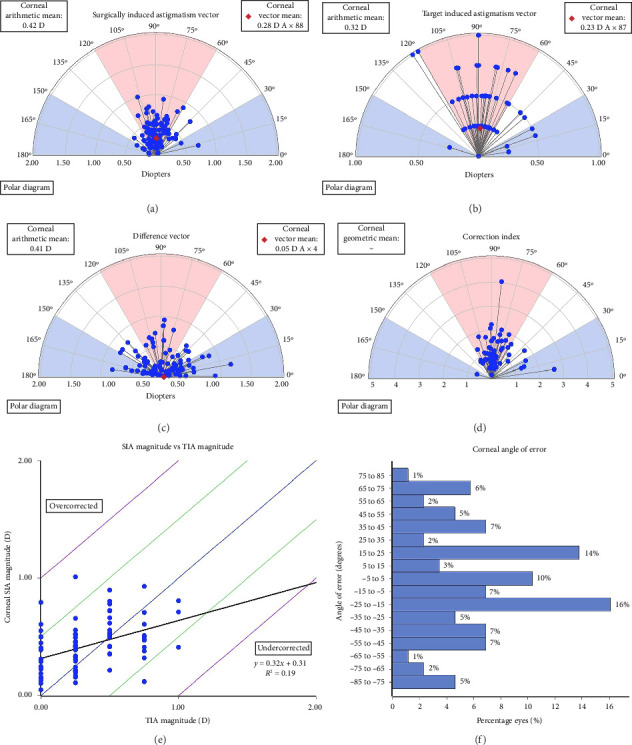
Single-angle polar plots at 6 months postoperative (a) SIA; (b) TIA; (c) DV; (d) CI; (e) SIA vs TIA; (f) Corneal angle of error.

At postoperative time of 1 w and 6 m, the SIA were −0.41 D ∗ 89 (mean −0.52 D) and −0.28 D ∗ 88 (mean −0.42 D), the TIA were −0.25 D ∗ 87 (mean −0.43 D) and −0.23 D ∗ 87 (mean −0.32 D), the correlation between TIA and SIA were *y* = 0.44*x* + 0.33, *R*^2^ = 0.24 and *y* = 0.32*x* + 0.31, *R*^2^ = 0.19. The DV were −0.16 D ∗ 2 (mean −0.50 D) and −0.05 D ∗ 4 (mean −0.41 D). The postoperative CI were all greater than 1, indicating mild overcorrection. Notably, the effect of overcorrection was more pronounced at 1 w postoperatively compared with 6 m. The IOS were 1.56 and 4.6 (Figures 5, 6, 7).

## 4. Discussion

The effectiveness of ICL implantation is closely linked to the postoperative residual diopter. Efforts should be made to avoid the situation of undercorrection, overcorrection, and residual astigmatism during the postoperative time. Postoperative astigmatism results from the vector sum of uncorrected or undercorrected preoperative astigmatism, as well as astigmatism caused by SIA, ICL eccentricity, and tilt. Among these factors, uncorrected or undercorrected preoperative astigmatism has the greatest impact on vision. The principle of astigmatism correction involves correcting preoperative astigmatism, controlling SIA, and minimizing astigmatism caused by ICL eccentricity and tilt. Selecting an appropriate ICL size can prevent ICL eccentricity and tilt, thereby correcting preoperative astigmatism and controlling SIA especially critical.

TICL implantation effectively corrects preoperative astigmatism and is commonly used as the preferred correction method for patients with myopia and astigmatism. Particularly for myopic patients with high astigmatism, by controlling the SIA to avoid the introduction of new astigmatism, the postoperative outcomes of TICL implantation can be achieved satisfactorily. Both SIA and postoperative TICL rotation can influence the effect of astigmatism correction, with TICL rotation being a frequent postoperative complication. Research [11] indicates that the TICL with a low vault increases the rotation risk. For optimal stability to TICL, TICL size should be carefully considered to ensure an appropriate postoperative vault. Zhu et al. [12], Damho et al. [13], and Hashem et al. [14] reported that the TICL exhibited mild rotation at postoperative 1 year, and the average rotation angles were 2.75 ± 2.04°, 2.4 ± 3.8°, and 2.68 ± 2.11°, respectively. This rotation was significantly correlated with the fixed angle during TICL implantation and the size of the TICL. Compared with TICL with a fixed angle < 5°, Zhu et al. [12] and Mori et al. [15] discovered that TICL with a fixed angle > 10° is significantly more likely to experience large-scale rotation, with the probability of rotation being 6.02 times. TICL with a fixed angle between 5° and 10° has a rotation probability that is 4.24 times. The fixed angle of the TICL is closely related to the position of the TICL haptics [11], which is affected by both the size of the TICL and the anatomy of the ciliary sulcus [16]. In situations where a ciliary cyst or an iris tumor occurs in the ciliary sulcus, the risk of TICL rotation increases [17]. Myopic patients with low to moderate astigmatism, who have abnormal ciliary sulcus morphology or large TICL fixed angles, are more likely to experience poor or unstable astigmatism correction postoperatively.

SIA is inevitable in intraocular surgery, and by minimizing or utilizing it, we can achieve postoperative residual astigmatism levels that approach 0 D, significantly increasing patient outcomes. Most myopic patients with low to moderate astigmatism can achieve good vision with either uncorrected or partially corrected astigmatism [3, 18]. Choosing the ICL combined with personalized SIA can help offset or reduce the original astigmatism, leading to better postoperative outcomes. This approach can also prevent issues such as the postoperative rotation of the lens. SIA for astigmatism correction is widely utilized in myopic patients with low to moderate astigmatism who are undergoing cataract surgery [4–7]^.^ These methods include techniques such as corneal ablation, the limbal relaxing incision (LRI), the clear corneal incision (CCI), the opposite clear corneal incision (OCCI), the arcuate keratectomy (AK) (with or without femtosecond laser assistance), and other methods [5]. LRI is particularly favored in clinical practice due to its cost-effectiveness and convenience. Personalized SIA is made by a full-layer corneal incision to make a personalized SIA while implanting an ICL, this method can correct CA and minimize multiple corneal injuries. This corneal incision is considered a special CCI at the corneal steep axis, which is located 1 mm inside the corneal limbus. The effectiveness of astigmatism correction depends on several factors, including the position, depth, length, structure, and precision of the corneal incision [19]. The corneal incisions of ICL implantation measure between 3.0 and 3.2 mm, while the SIA is generally approximately 0.5 D. Postoperative residual astigmatism tends to be more favorable with steep-axis corneal incisions than with flat-axis incisions [8, 9]. Additionally, personalized SIA can be up to 3.0 D [5].

Astigmatism typically refers to regular astigmatism, where CA has the most significant effect [20]. If the axial direction of the RA and the CA is less than 20° and the CA is greater than or equal to the RA, then the CA can be assumed to be equivalent to RA [21, 22]. The CA is the vector sum of anterior corneal astigmatism (ACA) and posterior corneal astigmatism (PCA), which is highly positively correlated with RA and is the main component and determining factor of RA [20, 23, 24]. Owing to the greater significant contribution of the ACA to astigmatism correction than the PCA, the CA is often assumed to be the ACA in refractive surgery. When the axial difference between the RA and CA is less than 15°, the corrective effect of the CA almost matches that of the RA [21, 22]. It is essential for accurately determining the size and axis of the CA. When the RA is influenced primarily by the CA, the closer the alignment of the CA value and axis to the RA, the better outcomes with the personalized SIA correction. In contrast for astigmatism dominated by ORA, simple corneal treatment provides no better correction effect than TICL. The postoperative changes in corneal morphology typically stabilize within 1 week to 3 months, and the effectiveness of astigmatism correction may gradually decrease or become insufficient [25, 26]. The astigmatism in most patients is regular astigmatism, which is influenced by factors such as eyelid coverage and has a relatively minor impact on visual acuity. Moderate correction of the regular CA can effectively address RA. With increasing age, regular astigmatism may progress to irregular or oblique astigmatism [23, 24, 27, 28]. A small amount of residual regular astigmatism typically does not affect postoperative vision and can help counterbalance the changes in RA associated with aging.

SIA may lead to axial deviations in postoperative astigmatism, with a portion of the-rule astigmatism potentially converting into oblique or against-the-rule astigmatism, which can compromise postoperative visual quality. Studies [29] have demonstrated a significant negative correlation between the absolute magnitude of low astigmatism and objective visual quality. For astigmatism of the same diopter, oblique and against-the-rule astigmatism exert a greater adverse effect on visual performance compared to the-rule astigmatism. Astigmatism less than 0.5 D generally does not significantly affect visual quality. Using SIA to correct astigmatism, the reduction in the absolute magnitude of astigmatism has a more significant influence on visual quality compared to the alteration in the axial orientation of astigmatism. Our study revealed that the corrective effect of SIA tends to diminish to varying degrees over time. In other words, certain cases of oblique or against-the-rule astigmatism that occur postoperatively may revert to the preoperative astigmatism axis status. Consequently, the influence of axial changes on the visual quality of astigmatism postoperatively appears to be relatively minor compared to astigmatism diopter changes.

Corneal incisions can affect light efficiency, leading to alterations in the surface asymmetry index (SAI) and corneal aberrations. HOAs, mainly spherical aberration (causing glare and halo) and coma aberration (resulting in blur and ghosting), significantly affect visual quality. Additionally, SIA can influence corneal aberrations by altering corneal morphology. The corneal incision was located 1 mm within the corneal limbus, which means that the changes in corneal morphology and regularity are minimal. As a result, there was no significant increase in corneal aberration. Postoperative symptoms that may affect visual quality—such as shadows, halos, glare, and decreased contrast sensitivity—tend to be relatively mild [30]. Pérez-Vives et al. [31] reported that the implantation of ICL introduced negative spherical aberrations without increasing other aberrations. The variation in spherical aberration is directly related to the refractive power of the ICL lens and is positively correlated with pupil size. While ICL implantation with different refractive powers did not significantly affect HOAs at a 3-mm pupil diameter, there was a significant increase in HOAs at a 4.5-mm pupil diameter. The positive spherical aberration in the Artisan and Artiflex lenses significantly increases at a 6-mm pupil compared with the EVO-ICL lens, with a more pronounced increase observed in the Artisan lens [32]. Kim et al. [33] mentioned that the ICL lens and corneal incisions can lead to HOAs. The ICL lenses affect primarily spherical aberration, while various corneal incisions mainly influence coma aberration. Dan et al. [30] reported no statistically significant difference in SIA and corneal aberration caused by CCI and limbus tunnel incision. Hashemian et al. [34] demonstrated that both the implantation of ICL and TICL can lead to negative spherical aberration. This phenomenon occurs due to the difference in how light is focused around the edges compared with the center of the ICL lens, resulting in negligible HOAs. Although there is a variation in refractive power between the flat and steep axes of the TICL lens, the spherical aberration produced by ICL and TICL implantation is not significantly different, and neither lens causes changes in other types of aberrations. Du et al. [35] demonstrated that TICL implantation does not influence CA or HOAs or increase total coma aberrations. Wang et al. [36] found that the CCI location had an impact on the SIA and the HOAs. Specifically, the superior CCI affected the clover aberration, with a positive correlation for the CCI angle and a negative correlation for the CCI width to SIA. Additionally, the temporal CCI affected horizontal coma, with no significant relationship with the SIA for the CCI angle and width. Adjusting the CCI angle and width is necessary to achieve an ideal personalized SIA. This can influence the effectiveness of astigmatism correction, while residual CA affects corneal aberration. At postoperative time, we observed a significant reduction in LOA and vertical aberration within 6 mm of the cornea. However, there was an increase in horizontal aberration, HOAs, and spherical aberration. This suggests that the changes in LOA and coma were caused by SIA, leading to a decrease in CA and an increase in HOAs. The changes in spherical aberration were mainly due to the implantation of the ICL lens.

Our research still has several limitations. Firstly, this study is a retrospective self-controlled study and lacks a control group. Secondly, it is a single-center study with a relatively small sample size. Finally, our evaluation of visual quality is based solely on visual acuity and corneal aberrations, which means that the evaluation indicators are not comprehensive enough. In the future, we should consider incorporating the visual quality scale and total eye aberration into our assessments to enhance the evaluation of postoperative visual quality.

## 5. Conclusion

ICL combined with personalized SIA can yield effective surgical outcomes for myopic patients with moderate to low astigmatism, without causing significant changes in corneal aberration. The effect of SIA on astigmatism correction is most pronounced within postoperative 1 week and tends to stabilize at postoperative 6 m. Partial residual astigmatism has a minimal impact on postoperative vision and can neutralize age-related changes in astigmatism in the entire eye.

## Figures and Tables

**Figure 1:**
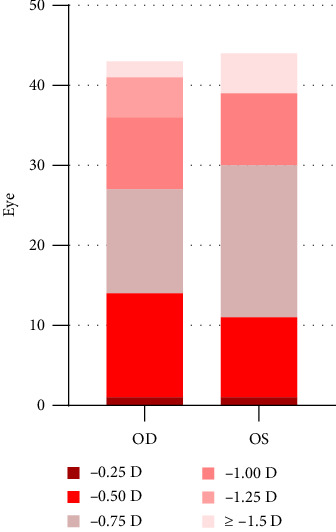
Preoperative cylindrical refractive error distribution.

**Table 1:** Preoperative characteristics of eyes with moderate to low astigmatism and myopia patients.

Variables	*N*	Age (y)	Sph (D)	Cyl (D)	SE (D)	IOP (mmHg)	Pupil (mm)	ECD (cells/mm^2^)	ACD (mm)	WTW (mm)	Eye axis (mm)	ICL size (mm)
Value	87 (43OD/44OS)	27.84 ± 4.95	−7.47 ± 2.61	−0.83 ± 0.35	−7.89 ± 2.65	15.95 ± 3.25	5.06 ± 0.79	2628.83 ± 207.81	3.23 ± 0.23	11.60 ± 0.34	26.39 ± 1.23	12.56 ± 0.26
Range (min, max)		19, 42	−18.00, −3.00	−2.25, −0.25	−18.75, −3.38	7.5, 22.1	3.06, 6.92	2137, 3053	2.83, 4.02	10.9, 12.4	24.06, 31.26	12.1, 13.2

*Note:* Sph = spherical refractive error; Cyl = cylindrical refractive error; D = diopter; ECD = endothelial cell count. Data are mean ± SD unless otherwise indicated.

Abbreviations: ACD = anterior chamber depth, IOP = intraocular pressure, SE = spherical equivalent, WTW = white-to-white.

**Table 2:** Visual acuity and diopter of preoperative versus postoperative 1 w and 6 m.

Variables	*n*	Preop				Postop 1 w			Postop 6 m		
		CDVA	EsCDVA	RA (D)	CA (D)	UDVA	RA (D)	CA (D)	UDVA	RA (D)	CA (D)
Value	87	0.95 ± 0.10	0.87 ± 0.14	−0.83 ± 0.35	−1.24 ± 0.45	0.95 ± 0.11	−0.40 ± 0.28	−0.86 ± 0.41	0.98 ± 0.08	−0.51 ± 0.32	−0.97 ± 0.47
Range (min, max)		0.6, 1.0	0.3, 1.0	−2.25, −0.25	−2.50, −0.3	0.5, 1.0	−1.50, 0	−2.00, −0.10	0.5, 1.0	−1.75, 0	−2.20, 0

*Note:* EsCDVA = estimated corrected distance visual acuity; RA = ocular refractive astigmatism. Data are mean ± SD unless otherwise indicated.

Abbreviations: CA = corneal astigmatism, CDVA = corrected distance visual acuity, UDVA = uncorrected distance visual acuity.

**Table 3:** Corneal aberrations of preoperative versus postoperative 1 w and 6 m using generalized estimated equation (GEE) analysis.

Variables (μm)	Total	LOA	HOA	Vertical coma	Horizontal coma	Spherical aberration
Preop	1.425 ± 0.363	1.374 ± 0.368	0.357 ± 0.101	−0.105 ± 0.140	−0.017 ± 0.145	0.157 ± 0.082
Postop						
1 w (vs. preop)	1.255 ± 0.373	1.166 ± 0.398	0.429 ± 0.106	−0.100 ± 0.131	−0.026 ± 0.133	0.168 ± 0.079
*P*	< 0.001	< 0.001	< 0.001	0.605	0.623	0.262
6 m (vs. preop)	1.287 ± 0.440	1.218 ± 0.451	0.392 ± 0.099	−0.078 ± 0.139	−0.016 ± 0.138	0.172 ± 0.080
*p*	< 0.001	< 0.001	0.019	0.016	1.000	0.163
1 w vs 6 m						
*p*	1.000	0.524	0.003	1.000	0.446	1.000

*Note:* Data are mean ± SD unless otherwise indicated.

Abbreviations: HOA = high-order aberration, LOA = low-order aberration.

## Data Availability

All the data generated or analyzed during this study are included in this article. The data that support the findings of this study are available from the corresponding author upon reasonable request.
